# Intergenerational Mobility of Earnings in China

**DOI:** 10.21203/rs.3.rs-2486612/v1

**Published:** 2023-01-24

**Authors:** yanmin wang, jing jin

**Affiliations:** Anhui University of Finance and Economics; Anhui University of Finance and Economics

**Keywords:** Intergenerational Earnings Mobility, Inequality of Opportunity, Minimum Distance Estimation, Earnings Dynamics, J62, C81, D31

## Abstract

Due to dataset limitations, existing studies on China’s intergenerational income mobility are unreliable. Using longitudinal data from the Chinese Health and Nutrition Survey, this study applied a modified version of the Zimmerman [Zimmerman DJ (1992) Regression toward mediocrity in economic stature. Am Econ Rev 82(3):409–429] model and estimated intergenerational earnings mobility based on a complete model with covariance restrictions. The new estimate demonstrates that intergenerational earnings elasticity in China is 0.54, a rather higher level relative to most developed countries.

## 1. Introduction

China’s economic growth over the past 40 years was an amazing global event. However, the other side of this coin is that China’s income inequality has increased sharply and is close to that of the US (Piketty et al. 2019). This outcome increased academic attention on income inequality with the aim of understanding the extent of income disparities. A broad consensus is that the income disparities in China, measured with the Gini coefficient, are very close to those of the US, a country with one of the highest inequality among developed countries (Cheng 2007; Yang and Yang 2015).

Inequality of opportunity has become a tremendously salient issue for policy makers in China. In the three recent National Congresses of the Communist Party of China, the party called for the government to improve equality of opportunity. Equality of opportunity is measured by intergenerational mobility. Given the widespread concern about intergenerational mobility, it is astonishing that there is no any official document concisely presents the intergenerational elasticity (IGE) in earnings between fathers and sons, which measures the extent that individuals inherit their parents’ position in the income distribution. Although several studies were conducted, these are of no help for ascertaining the degree of intergeneration mobility in China. Compared to the research in the US and Sweden, most studies on China’s intergeneration mobility did not use an intergenerational sample from longitudinal data, but rather depended on cross-sectional or retrospective data on the incomes of parents at an earlier date (Wang 2005; Gong et al. 2012; Chen 2013; Deng et al. 2013; Fan 2016). With the application of longitudinal data, especially the Chinese Health and Nutrition Survey (CHNS), a limited number of studies used short panels to estimate intergenerational mobility (Yao and Zhao 2006; Labar 2007; Han 2010; Yan and Deng 2021). The estimates of the level of mobility in China as a whole are below 0.5, with the exception of Yao and Zhao’s (2006) IGE estimates, which imply much lower levels of intergenerational mobility.

However, all the longitudinal datasets except the CHNS do not have a period long enough to conduct intergenerational analysis. Even the CHNS is also not a perfect data source, like the Panel Survey of Income Dynamics (PSID) in the US. Firstly, it is not nationally representative survey. In the last waves, the CHNS covered 15 provinces among 31 provinces in the mainland China, excluding Hong Kong, Taiwan and Macau. Secondly, the survey does not follow family members as they formed new households. As a result, parents and sons in the sample for intergenerational analysis is in co-residence condition. Few authors attempted to address the problems; hence, we should be cautious interpreting their conclusions.

In China’s context, the CHNS is not too bad. The problems because of sampling design are less than expected. Indeed, the CHNS covers most provinces east of the Hu Line. This is significant because China’s population is distributed unevenly, with about 94% living east of the Hu Line on about 43% of China’s land area (Chen et al. 2016). Additionally, the selection bias induced by the co-residence condition is far less serious than was earlier believed. In China, if a family only has one son, the parent and son will live in the same household until the parent dies. If a family has more than two sons, then the families will not be broken up until the sons marry. That is, parents and sons will live in the same households for a long time. Nevertheless, if we can collect as much information as possible about adult earnings, the risk for selection bias will be reduced.

In this study, we extend the Zimmerman’s (1992) econometric model and estimate the IGE based on data from the CHNS. The results contain strong evidence that intergenerational earnings elasticity is 0.54 or even higher, depicting a much less mobile society than suggested by earlier studies.

The rest of this paper is organized as follows. Section 2 briefly review the relevant literature on intergenerational earnings mobility. Section 3 describes the CHNS data and data processing. In Section 4, we develop an econometric models and discuss the estimation methods. Section 5 presents the empirical results, and Section 6 concludes the main findings.

## 2. Literature Review

The most commonly measure for intergenerational income mobility is the elasticity of lifetime income between fathers and sons; that is, the estimate of *β* obtained from the following regression:

(1) $$y_{i}^{c}=\alpha +\beta y_{i}^{p}+\varepsilon_{i},$$

where $y_{i}^{p}$ represents the permanent earnings component in the parent’s generation, and $y_{i}^{c}$ represents the permanent earnings component of the child’s generation. In the economics literature, the permanent component is the expectation of the log of individual earnings in a given period (*y*_*it*_):

(2) $$y_{it}=y_{i}+v_{it},$$

where *v*_*it*_ represents the error term. In a linear errors-in-variables model, the ordinary least square (OLS) estimator of *β* is downward biased if there is error in independent variable (Greene 2018).

Since 1990s, the intergenerational mobility literature, especially on measurement of the IGE, has flourished. The consensus among the social scientists is that sons’ earnings should lag one generation, at least 15 years and even more, behind fathers’ earnings to control for life cycle biases. Most influential studies in the US were based on survey data. A landmark study by Solon (1992), using PSID data, showed that the estimate using a single year of income were heavily downward biased. Using up to the five-year average of the father’s earnings, Solon’s (1992) estimate is close to 0.4, largely higher than the estimates in previous studies. Using survey data matched to administrative data from Social Security Administration, Mazumder (2005) averaged fathers’ earnings over 16 years and yielded the estimate of IGE was greater than 0.6. Chetty et al.(2014) argued that Mazumder’s (2005) estimate is upward biased because the rate of imputing the missing data for parent income based on race and education is so higher that the estimate is analogous to instrumental variable estimator. Using federal income tax records, Chetty et al. (2014) estimated the IGE to be 0.34. Mazumder (2016) explained that the Chetty et al.’s (2014) estimate is so low because of the measurement error of the sons’ earnings. Hence, even in such large-scale settings, it is difficult to address the measurement error of the variables in linear models.

There should be a unified framework to address the measure error in both fathers’ earnings and sons’ earnings. Zimmerman (1992) made the earliest attempt. He assumed that *v*_*it*_ followed a first-order autoregressive process and estimated all the parameters in a completed model with minimum distance estimation (MDE). Altonji and Dunn (2012) assumed *v*_*it*_ followed a white noise or second-order moving average process and estimated the intergenerational correlation with the method of moments. The Zimmerman’s (1992) model is better because the estimation results include the parameters that reflect both income inequality and intergenerational mobility. Such econometric model meets the requirement of theoretical models on social determinants of persistent inequality (Durlauf and Seshadri 2018; Fogli and Guerrieri 2019). The difficulty is that classic MDE is incapable of handling unbalanced data: every person has different time horizon, and parent and son in a family have different time horizon.

However, there has been process in recent years. Dickens (2000) developed a procedure to estimate and infer parameters in univariate variance component models with unbalanced panel data. Blundell et al. (2008) extended the procedure to examines the link between income and consumption inequality. We utilize their method to estimate China’s IGE of earnings. The method enables all available information from randomised participants to be included, as well as from dropouts. Thus, all the paired samples with adult earnings are available, and our sample size is larger.

## 3. Data

The CHNS is an international collaborative project between University of North Carolina at Chapel Hill and the Chinese Centers for Disease Control and Prevention (CCDC). The survey has been conducted aperiodically since 1989. Nine additional panels were collected in 1991, 1993, 1997, 2000, 2004, 2006, 2009, 2011, and 2015. The number of families surveyed increased from 3,795 in 1989 to 5,892 in 2015. The first wave of the surveys covered nine provinces. A new province was added in 1997 when another province quit the survey, and this province returned the survey in 2000. The CHNS have included three mega-cities since 2011 and added three provinces in 2015. The files for the 1989–2015 surveys are available from the CHNS website for public use.

As a dual economy, the labor income is composite. It consists of individual income, including wage income, bonuses, commissions, and the portion of family income from business, farming, fishing, gardening, and livestock. The labor income variable is in 2014 RMB as measured by the consumer price index.

The sample is restricted to adult men aged 16 – 65 years reporting a positive annual income, excluding students at school. The fathers are the male heads of households. The sons are not required to be biological sons. If more than one son from the same family meets all the above restrictions, then we retain only the oldest son for the analysis.

It should be pointed out that the sample includes father-son pairs living together for at least one round. If parents and sons are not in co-residence conditions through all rounds, then we cannot capture such families. Thus, the main sample comprises 1, 999 families with 8, 517 person-year observations of fathers and 5, 290 person-year observations of sons. The summary statistics of the labor income variable is shown in Table 1. There are signs that the mean log earnings is rising and the standard deviation is stable. Hence, a demeaning procedure (subtracting the mean from the data) should allow for year effects.

## 4. Econometric Models and Estimation Methods

### 4.1 Models

In the transitory component in equation (2), we assume that *v*_*it*_ follows an AR(1) process:

(3) $$v_{it}=\rho v_{it-1}+\xi_{it}\text{, }$$

where *ξ*_*it*_ represents white noise. The serial correlation coefficient, *ρ*, represents the rate of deterioration of the effects of random shocks that persist for many years. We obtain the process of *v*_*it*_ recursively:

(4) $$v_{it}=\overset{t-1}{\underset{k=0}{\sum }}\rho^{k}\xi_{it-k}+\rho^{t}v_{i0}$$

where *v*_*i*0_ represents the initial shock. The moments implied by equations (1)–(4) are

(5) $$Cov\left(y_{it}^{c},y_{is}^{c}\right)=Var\left(y_{i}^{c}\right)+\rho_{c}^{\left(t-s\right)}Var\left(y_{is}^{c}\right),\;t\geq s$$

(6) $$Cov\left(y_{it}^{p},y_{is}^{p}\right)=Var\left(y_{i}^{p}\right)+\rho_{p}^{\left(t-s\right)}Var\left(v_{is}^{p}\right),\;t\geq s$$

(7) $$Var\left(v_{it}^{c}\right)=Var\left(\xi_{it}^{c}\right)\times \left(1+\rho_{c}^{2}+\rho_{c}^{4}+\cdots +\rho_{c}^{2t-2}\right)+\rho_{c}^{2t}Var\left(v_{i0}^{c}\right)$$

(8) $$Var\left(v_{it}^{p}\right)=Var\left(\xi_{it}^{p}\right)\times \left(1+\rho_{p}^{2}+\rho_{p}^{4}+\cdots +\rho_{p}^{2t-2}\right)+\rho_{p}^{2t}Var\left(v_{i0}^{p}\right)$$

(9) $$Cov\left(Y_{it}^{c},Y_{is}^{p}\right)=\beta Var\left(Y_{i}^{p}\right)+Cov\left(v_{it}^{c},v_{is}^{p}\right).$$

where *Var*(·) and *Cov*(·, ·) represent cross-sectional variance and covariance respectively. Our model extends Zimmerman’s (1992) specification. He assumed no correlation in transitory income between fathers and sons. This assumption is reasonable if fathers and sons are not observed at the same time. However, every survey in the CHNS always contains the earnings data for both fathers and sons; therefore, the innovations for transitory income and initial shock between parents and sons should be correlated. For sake of simplicity, we assume that the covariance of the innovations between fathers and sons are time-invariant; that is, $Cov\left(\xi_{i-k}^{c},\xi_{i-k}^{p}\right)=Cov\left(\xi_{i}^{c},\xi_{i}^{p}\right)$; thus,

(10) $$\begin{array}{l} Cov\left(v_{is}^{c},v_{is}^{p}\right)=\overset{s-1}{\underset{k=0}{\sum }}{\left(\rho_{c}\rho_{p}\right)}^{k}Cov\left(\xi_{i}^{c},\xi_{i}^{p}\right)+{\left(\rho_{c}\rho_{p}\right)}^{s}Cov\left(v_{i0}^{c},v_{i0}^{p}\right)\\ Cov\left(v_{it}^{c},v_{is}^{p}\right)=\rho_{c}^{t-s}Cov\left(v_{is}^{c},v_{is}^{p}\right),t\geq s\\ Cov\left(v_{it}^{c},v_{is}^{p}\right)=\rho_{p}^{s-t}Cov\left(v_{it}^{c},v_{it}^{p}\right),t≺s \end{array}.$$

We refer to the complete statistical model in (5)–(10) as our “base model”. It goes beyond earlier models by allowing for correlation in transitory components between fathers and sons. The parameters in our model are $\left\{Var\left(y_{i}^{c}\right),Var\left(y_{i}^{p}\right),\text{var}\left(\xi_{i}^{c}\right),Var\left(\xi_{i}^{p}\right),\rho_{c},\rho_{p},Var\left(v_{i0}^{c}\right),Var\left(v_{i0}^{p}\right),\beta ,Cov\left(\xi_{i}^{c},\xi_{i}^{p}\right),Cov\left(v_{i0}^{c},v_{i0}^{p}\right)\right\}$.

### 4.2 Estimation Methods

Our measure of *y*_*it*_ was defined as the deviation of observed log earnings from the mean. We follow the demeaning procedure from the most recent literature and adjust for year, age and region effects on average earnings in a pooled regression.

Next, we estimate the complete model using MDE, which minimizes the distance between the observed sample moments in the data, *m*, and the corresponding population moments in the model, *f*(*θ*). *θ* is the parameters in our model. The objective function is formulated below.

(11) $$D=(m-f(\theta ){)}^{\prime }W(m-f(\theta ))\text{, }$$

where *W* is the identity matrix as the weighting matrix to reduce small-sample bias (Altonji and Segal 1996). The variance-covariance matrix of $\overset{⌢}{\theta }$ is

(12) $$Var(\overset{⌢}{\theta })={\left(G^{\prime }WG\right)}^{-1}G^{\prime }\text{WVWG}{\left(G^{\prime }WG\right)}^{-1},$$

where $G={\frac{\partial f(\theta )}{\partial \theta }|}_{\theta =\overset{⌢}{\theta }}$ is the Jacob matrix of *f*(*θ*) evaluated at $\overset{⌢}{\theta }$ and *V* is the variance-covariance matrix of *m*.

## 5. Empirical Results

The first part of this section presents the estimate of IGE based on MDE. The second part is the results of robust analysis.

### 5.1 MDE Results

We applied MDE of the complete model laid out in Section 4. The first column in Table 2 shows the resulting estimates of the base model, which allows for the correlation of the earnings’ innovations between fathers and sons. For comparison, the last two columns report the results of Restricted Model I and Restricted Model II, which follow Zimmerman’s (1992) specification and a common econometric specification, respectively. Both of models assume that transitory earnings between fathers and sons are uncorrelated. That is, $Cov\left(v_{it}^{c},v_{is}^{p}\right)=0$. Additionally, Restricted Model II specifies the variance of initial shock, *Var*(*v*_*i*0_), is zero. The last two rows in Table 2 present the goodness-of-fit estimates. We found that the more comprehensive the model was, the better the model became. Both the *χ*^2^ statistic and the value of objective function, $D(\hat{\theta })$, suggest that the data were best fitted by the base model.

We note that the magnitudes of the estimates of $Cov\left(\xi_{i}^{c},\xi_{i}^{p}\right)$ and $Cov\left(v_{i0}^{c},v_{i0}^{p}\right)$ are fairly large and the signs are positive. Thus, Restricted Models I and II suffer from omitting variables bias, and the IGE estimates are more than 30% higher than the estimate in the base model.

In the base model, the IGE estimate is about 0.54, which is close to that of the US (Mazumder 2005). What does an IGE of 0.54 imply? From a long-run perspective, if a family is in poverty, then it will take the offspring of this family 5 generations (100 years) to escape poverty; thus, China has low intergenerational mobility relative to most developed countries.

Table 2 also presents the parameters on income dynamics, which are similar between fathers and sons. The simulation with equations (5)–(8) indicates that the transitory component of earnings account for 80 percent of the total variance. The result echoes Xu and Zhu’s (2011) findings, suggesting that most of the earnings inequality in China is due to high inequality in the transitory component of earnings. In addition, the permanent share was approximately 0.5 in the US (Mazumder 2001). Therefore, the earnings process is obviously different between China and the US.

### 5.2. Sensitivity Analysis

In this case, we consider two variants of the Table 2 estimation for the base model. The first changes the time horizon and decreases the main sample. After removing first or last one/two waves, we obtained 4 subgroup samples: 1989–2011, 1989–2009, 1991–2015, and 1993–2015. Table 3 presents the results. The estimates of *β* do not change much and range from 0.568 (SE = 0.115) when the sample period is 1993–2015 to 0.636 (SE = 0.111) when sample period is 1989–2009.

Next, we exclude persons with only a single observation. Doing so shrinks the sample to 1, 085 father-son pairs and produces $\overset{⌢}{\beta }=0.689$ (SE = 0.106).

Despite the variation in results, all the estimates are distinctly above 0.5. Corresponding to China’s Gini coefficient obtained from previous studies, we can conclude that China in the last 30 years became a country with high inequality and low intergenerational mobility.

## 6. Conclusions

Previous studies in China reported inconsistent estimates of the IGE given the data limitations. Based on an intergenerational data from the CHNS, this study estimated intergenerational earnings mobility with a complete model with covariance restrictions. The results indicate that the IGE is 0.54. Combined with the Gini coefficient, we can conclude that China has a relatively higher outcome and chance of inequality. The empirical results also verify the validity of the “Great Gatsby curve” (Corak 2013).

Our model extends Zimmerman’s (1992) specification and has a practical value for small-scale longitudinal data, in which the earnings of fathers and sons are always correlated. The estimation method for unbalanced panel data is not new, though we offer the first application in measuring intergenerational mobility. Depending on the econometric model and estimation method, we have instruments to collect as much earnings information as possible in a short period. The selection bias induced by co-residence conditions will be greatly reduced. This method could be especially useful to developing countries, which is in lack of longitudinal data with long follow-up periods and large scale.

However, this study has two limitations. First, the relatively small scale of the CHNS does not allow us to account for life cycle bias. However, numerous studies call this assumption into question (e.g., Guvenen 2009; Moffitt and Gottschalk 2012). Another limitation is that we neglect the effect of co-residence condition. It is difficult to ascertain the impact of co-residence on the estimates of intergenerational mobility. To control for co-residence bias, survey design, following children from original family to their new family, is better than econometric model, such as Heckman sample selection model. Further research into China’s intergenerational mobility will require the introduction of better longitudinal data, like the PSID.

## Figures and Tables

**Table 1:** Sample Characteristics

		Fathers			Sons	
Year	Log earnings (price in 2014)			Log earnings (price in 2014)		
	N	mean	Standard deviation	N	mean	Standard deviation
1989	1303	8.081	1.052	523	7.698	1.071
1991	1287	8.111	0.981	603	7.735	1.042
1993	1100	8.126	1.063	601	7.896	1.199
1997	971	8.427	1.030	627	8.316	1.099
2000	966	8.557	1.141	705	8.534	1.236
2004	714	8.749	1.101	392	8.784	1.145
2006	661	9.044	1.148	344	9.255	1.081
2009	587	9.401	1.086	443	9.635	1.046
2011	575	9.577	1.091	472	9.909	0.918
2015	353	9.946	1.309	580	10.371	1.099

**Table 2:** Minimum Distance Estimation

Parameter	Base Model	Restricted Model I	Restricted Model II
$Var\left(Y_{i}^{p}\right)$	0.207***(0.02)	0.213***(0.02)	0.214***(0.02)
*ρ*_*p*_	0.427***(0.04)	0.37***(0.04)	0.366***(0.04)
$Var\left(v_{i0}^{p}\right)$	0.869***(0.062)	0.875***(0.062)	
$Var\left(\xi_{i}^{p}\right)$	0.756***(0.038)	0.795***(0.036)	0.794***(0.034)
$Var\left(Y_{i}^{c}\right)$	0.17***(0.032)	0.177***(0.031)	0.178***(0.031)
*ρ*_*c*_	0.519***(0.048)	0.479***(0.051)	0.472***(0.051)
$Var\left(v_{i0}^{c}\right)$	0.87***(0.102)	0.879***(0.102)	
$Var\left(\xi_{i}^{c}\right)$	0.691***(0.043)	0.725***(0.043)	0.726***(0.042)
*β*	0.544***(0.11)	0.7***(0.105)	0.699***(0.104)
$Cov\left(\xi_{i}^{c},\xi_{i}^{p}\right)$	0.282***(0.078)		
$Cov\left(v_{i0}^{c},v_{i0}^{p}\right)$	0.22***(0.025)		
$D(\hat{\theta })$	2.724	3.421	3.426
*χ* ^2^	379.448	460.128	460.3

Source: Authors’ computation.

Note: standard errors in parentheses.

***, **, and * denote 0.01, 0.05, and 0.10 rejection levels of significance, respectively.

**Table 3:** Robustness Analysis (smaller sample)

Parameter	1989–2011	1989–2009	1991–2015	1993–2015
$Var\left(Y_{i}^{p}\right)$	0.191***(0.019)	0.187***(0.018)	0.214***(0.022)	0.24***(0.025)
*ρ*_*p*_	0.485***(0.034)	0.468***(0.033)	0.445***(0.047)	0.397***(0.062)
$Var\left(v_{i0}^{p}\right)$	0.871***(0.061)	0.883***(0.061)	0.707***(0.064)	0.838***(0.076)
$Var\left(\xi_{i}^{p}\right)$	0.679***(0.032)	0.695***(0.031)	0.757***(0.043)	0.782***(0.05)
$Var\left(Y_{i}^{c}\right)$	0.177***(0.036)	0.179***(0.035)	0.198***(0.035)	0.221***(0.043)
*ρ*_*c*_	0.506***(0.051)	0.502***(0.048)	0.5***(0.061)	0.496***(0.081)
$Var\left(v_{i0}^{c}\right)$	0.865***(0.104)	0.868***(0.103)	0.797***(0.088)	1.114***(0.114)
$Var\left(\xi_{i}^{c}\right)$	0.73***(0.046)	0.695***(0.041)	0.698***(0.05)	0.661***(0.058)
*β*	0.629***(0.103)	0.636***(0.111)	0.592***(0.11)	0.568***(0.115)
$Cov\left(\xi_{i}^{c},\xi_{i}^{p}\right)$	0.265***(0.076)	0.27***(0.077)	0.337***(0.074)	0.446***(0.075)
$Cov\left(v_{i0}^{c},v_{i0}^{p}\right)$	0.242***(0.025)	0.228***(0.024)	0.2***(0.027)	0.176***(0.031)
$D(\hat{\theta })$	1.384	1.85	2.195	1.647
*χ* ^2^	223.697	305.841	291.774	226.981
No. of panels				

Source: Authors’ computation

Note: standard errors in parentheses.

***, **, and * denote 0.01, 0.05, and 0.10 rejection levels of significance, respectively.

**Table 4:** Robustness Analysis (repeated observations)

Parameter	Estimate	Parameter	Estimate
$Var\left(Y_{i}^{p}\right)$	0.196***(0.026)	$Var\left(Y_{i}^{c}\right)$	0.164***(0.035)
*ρ*_*p*_	0.45***(0.044)	*ρ*_*c*_	0.519***(0.049)
$Var\left(v_{i0}^{p}\right)$	0.876***(0.079)	$Var\left(v_{i0}^{c}\right)$	0.946***(0.116)
$Var\left(\xi_{i}^{p}\right)$	0.752***(0.047)	$Var\left(\xi_{i}^{c}\right)$	0.693***(0.047)
*β*	0.689***(0.106)	$D(\hat{\theta })$	2.795
$Cov\left(\xi_{i}^{c},\xi_{i}^{p}\right)$	0.297***(0.09)	*χ* ^2^	444.884
$Cov\left(v_{i0}^{c},v_{i0}^{p}\right)$	0.219***(0.028)		

Source: Authors’ computation

Note: standard errors in parentheses.

***, **, and * denote 0.01, 0.05, and 0.10 rejection levels of significance, respectively.

## Data Availability

CHNS data are free and available for registered users. CHNS files can be downloaded directly from the website (https://www.cpc.unc.edu/projects/china/dat a/datasets/data_downloads/).

## Appendix A. The moment of earnings covariance between fathers and sons

According to equation (1) – (3), the moment of earnings covariance between fathers and sons is

(A1)
$$\begin{array}{l} Cov\left(y_{it}^{c},y_{is}^{p}\right)=Cov\left(y_{i}^{c}+v_{it}^{c},y_{i}^{p}+v_{is}^{p}\right)\\ =Cov\left(y_{i}^{c},y_{i}^{p}\right)+Cov\left(y_{i}^{c},v_{is}^{p}\right)+Cov\left(y_{i}^{p},v_{it}^{c}\right)+Cov\left(v_{it}^{c},v_{is}^{p}\right) \end{array}.$$

The permanent and transitory components between fathers and sons are orthogonal to each other; that is, $Cov\left(y_{i}^{c},v_{is}^{p}\right)+Cov\left(y_{i}^{p},v_{it}^{c}\right)=0$. Then we have the formulas:

(A2)
$$Cov\left(y_{it}^{c},y_{is}^{p}\right)=\beta Var\left(y_{i}^{p}\right)+Cov\left(v_{it}^{c},v_{is}^{p}\right)$$

and

(A3)
$$Cov\left(v_{it}^{c},v_{is}^{p}\right)=Cov\left(\rho_{c}^{t-s}v_{is}^{c},v_{is}^{p}\right)=\rho_{c}^{t-s}Cov\left(v_{is}^{c},v_{is}^{p}\right),t\geq s\;Cov\left(v_{is}^{c},v_{is}^{p}\right)=\overset{s-1}{\underset{k=0}{\sum }}{\left(\rho_{c}\rho_{p}\right)}^{k}Cov\left(\xi_{i-k}^{c},\xi_{i-k}^{p}\right)+{\left(\rho_{c}\rho_{p}\right)}^{s}Cov\left(v_{i0}^{c},v_{i0}^{p}\right).$$

This used the assumption that only the innovation of transitory component in the same period between fathers and sons are correlated, and the innovations are uncorrelated with the initial shock between fathers and sons, $Cov\left(\xi_{it-k}^{c},v_{i0}^{p}\right)=Cov\left(v_{i0}^{c},\xi_{is-k}^{p}\right)=0$.

In addition, we assume that the covariance of innovations between fathers and sons are time-invariant, $Cov\left(\xi_{it-k}^{c},\xi_{it-k}^{p}\right)=Cov\left(\xi_{i}^{c},\xi_{i}^{p}\right)$. As a result, we obtain the following formula:

(A4)
$$Cov\left(v_{is}^{c},v_{is}^{p}\right)=\overset{s-1}{\underset{k=0}{\sum }}{\left(\rho_{c}\rho_{p}\right)}^{k}Cov\left(\xi_{i}^{c},\xi_{i}^{p}\right)+{\left(\rho_{c}\rho_{p}\right)}^{s}Cov\left(v_{i0}^{c},v_{i0}^{p}\right)\;Cov\left(v_{it}^{c},v_{is}^{p}\right)=\rho_{c}^{t-s}Cov\left(v_{is}^{c},v_{is}^{p}\right),t\geq s\;Cov\left(v_{it}^{c},v_{is}^{p}\right)=\rho_{p}^{s-t}Cov\left(v_{it}^{c},v_{it}^{p}\right),t≺s$$

## Appendix B. Estimation Details

The CHNS comprises of 10 waves: 1989, 1991, 1993, 1997, 2000, 2004, 2006, 2009, 2011, and 2015. The two primary vectors of interest are

(B1)
$$y_{i}^{p}=\{\begin{array}{l} y_{i0}^{p}\\ \cdots \\ y_{i11}^{p}\\ \cdots \\ y_{i26}^{p} \end{array}\text{ and }y_{i}^{c}=\{\begin{array}{l} y_{i0}^{c}\\ \cdots \\ y_{i11}^{c}\\ \cdots \\ y_{i26}^{c} \end{array}.$$

In line with the vectors above, we define

(B2)
$$d_{i}^{p}=\{\begin{array}{l} d_{i0}^{p}\\ \cdots \\ d_{i11}^{p}\\ \cdots \\ d_{i26}^{p} \end{array}\text{ and }d_{i}^{c}=\{\begin{array}{l} d_{i0}^{c}\\ \cdots \\ d_{i11}^{c}\\ \cdots \\ d_{i26}^{c} \end{array},$$

where $d_{it}^{p}=1$ {$y_{it}^{p}$ is not missing} and $d_{it}^{c}=1$ {$y_{it}^{c}$ is not missing}.

Stacking observations on *y*^*p*^ and *y*^*c*^ (and *d*^*p*^ on *d*^*c*^) for each individual, we obtain the vectors

(B3)
$$y_{i}=\left(\begin{array}{c} y_{i}^{p}\\ y_{i}^{c} \end{array}\right)\text{ and }d_{i}=\left(\begin{array}{c} d_{i}^{p}\\ d_{i}^{c} \end{array}\right).$$

Now, we can derive

(B4)
$$m=\text{vech}\left\{\left(\overset{N}{\underset{i=1}{\sum }}y_{i}y_{i}^{\prime }\right)\text{\%}\left(\overset{N}{\underset{i=1}{\sum }}d_{i}d_{i}^{\prime }\right)\right\},$$

where % denotes an elementwise division, *vech*() transforms the square and typically symmetric matrix into a column vector, and only the lower half of the matrix is recorded. The vector *m* includes the estimates of $Cov\left(y_{it}^{p},y_{is}^{p}\right)、Cov\left(y_{it}^{c},y_{is}^{c}\right)和Cov\left(y_{it}^{c},y_{is}^{p}\right)$.

The variance-covariance matrix of *m* that can be used for inference is

(B5)
$$V=\left\{\overset{N}{\underset{i=1}{\sum }}\left[\left(m_{i}-m\right){\left(m_{i}-m\right)}^{\prime }\right]⊗\left(D_{i}D_{i}^{\prime }\right)\right\}\text{\%}DD^{\prime },$$

where $m_{i}=\text{vech}\left(y_{i}y_{i}^{\prime }\right)$, $D_{i}=\text{vech}\left(d_{i}d_{i}^{\prime }\right)$, $D=\text{vech}\left(\sum_{i=1}^{N}d_{i}d_{i}^{\prime }\right)$, and ⊗ denotes an element-wise product. The square roots of the elements in the main diagonal of *V* provide the standard errors of the corresponding elements in *m*.
